# Dispersion-less Kerr solitons in spectrally confined optical cavities

**DOI:** 10.1038/s41377-022-01052-8

**Published:** 2023-01-09

**Authors:** Xiaoxiao Xue, Philippe Grelu, Bofan Yang, Mian Wang, Shangyuan Li, Xiaoping Zheng, Bingkun Zhou

**Affiliations:** 1 Department of Electronic Engineering, Beijing National Research Center for Information Science and Technology, Tsinghua University, 100084 Beijing, China; 2 Laboratoire ICB, UMR 6303 CNRS, Université Bourgogne Franche-Comté, 21000 Dijon, France

**Keywords:** Solitons, Nonlinear optics

## Abstract

Solitons are self-reinforcing localized wave packets that manifest in the major areas of nonlinear science, from optics to biology and Bose–Einstein condensates. Recently, optically driven dissipative solitons have attracted great attention for the implementation of the chip-scale frequency combs that are decisive for communications, spectroscopy, neural computing, and quantum information processing. In the current understanding, the generation of temporal solitons involves the chromatic dispersion as a key enabling physical effect, acting either globally or locally on the cavity dynamics in a decisive way. Here, we report on a novel class of solitons, both theoretically and experimentally, which builds up in spectrally confined optical cavities when dispersion is practically absent, both globally and locally. Precisely, the interplay between the Kerr nonlinearity and spectral filtering results in an infinite hierarchy of eigenfunctions which, combined with optical gain, allow for the generation of stable dispersion-less dissipative solitons in a previously unexplored regime. When the filter order tends to infinity, we find an unexpected link between dissipative and conservative solitons, in the form of Nyquist-pulse-like solitons endowed with an ultra-flat spectrum. In contrast to the conventional dispersion-enabled nonlinear Schrödinger solitons, these dispersion-less Nyquist solitons build on a fully confined spectrum and their energy scaling is not constrained by the pulse duration. Dispersion-less soliton molecules and their deterministic transitioning to single solitons are also evidenced. These findings broaden the fundamental scope of the dissipative soliton paradigm and open new avenues for generating soliton pulses and frequency combs endowed with unprecedented temporal and spectral features.

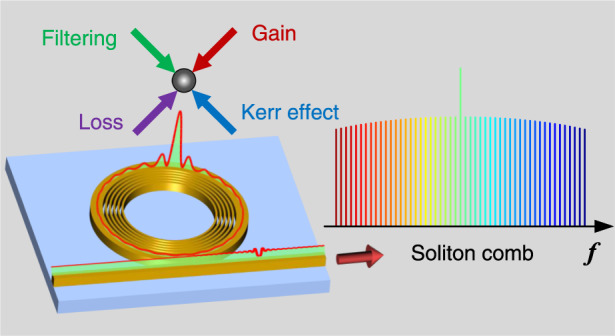

## Abstract (web summary)

A novel class of solitons, which builds up in spectrally confined optical cavities when dispersion is practically absent, is revealed both theoretically and experimentally, opening new avenues for generating soliton pulses and frequency combs endowed with unprecedented temporal and spectral features.

## Introduction

Soliton phenomenon has amazed and stimulated scientists since its first observation by John Scott Russell in 1834^1^ and led to many remarkable applications, particularly in optics^2,3^. The stationary pulsed solutions of the conservative nonlinear Schrödinger equation (NLSE) once appeared as a fascinating prospect for high-capacity optical communications^4^. The research was soon extended to the investigation of dissipative solitons in optical cavities for developing novel mode-locked laser architectures^5^ and Kerr frequency combs^6^. Dissipative solitons are sustained by the composite balance between dispersion and nonlinearity on the one hand and gain and loss on the other hand. In contrast to the conservative NLSE solitons, dissipative solitons feature frequency chirping, which reflects a necessary energy redistribution within the pulse as it propagates. The latter phenomenon increases the soliton diversity well beyond that available in Hamiltonian conservative systems. For instance, the mode locking of frequency-chirped bright laser pulses becomes accessible within the normal dispersion regime through an interplay involving spectral filtering^7–9^. Since it represents an elementary source of frequency chirping, the chromatic dispersion is considered as a key enabling physical effect in the generation of ultrashort pulses, acting either globally or locally on the cavity dynamics in a decisive way. For instance, some laser architectures, while they operate at a near-zero path-averaged dispersion, utilize a strong local dispersion to enhance the intracavity dynamics resulting, for instance, in a significant energy increase for the dispersion-managed propagating stretched pulses^10,11^. In other architectures, the local dispersion is combined with a lumped spectral filtering to access, within the laser cavity, a self-similar propagation evolution that becomes a robust attracting state^12^. Therefore, even if the net dispersion may be close to zero in the two architectures mentioned above, the local dispersion plays a major role in establishing the pulse dynamics. On the other hand, when the local second-order dispersion approaches zero, higher-order dispersion terms may become important and also enable soliton formation^13–17^. Although the existence of solitons in the absence of any chromatic dispersion has been suggested in the context of mode-locked lasers involving saturable absorbers^18,19^, no clear supportive results have ever been reported so far. Exploring such possibility is not only important for a broader understanding of the fundamental nonlinear dynamics of complex systems, but it is likely to enable novel ultrafast sources for a wide range of applications.

In this article, we present our findings concerning a novel category of dissipative optical solitons, which we call *dispersion-less solitons* as they arise from the interplay between dissipation and Kerr nonlinearity in the absence of chromatic dispersion. We base these findings on a well-known physical system, suitable for both theoretical modeling and experimental testing: the optically driven Kerr cavity^20–23^. Driving the Kerr cavity to new parameter regions, we uncover previously uncharted regimes in which ultra-flat spectrum, transform-limited pulse generation and superior energy scaling could be simultaneously achieved. We show that these dispersion-less solitons are closely related to the eigenfunctions of combined self-phase modulation (SPM) and spectral filtering effects, which asymptotically evolve toward an energy-conserved pulsed solution with the increase of the filter order. Such remarkable trend thus reveals the existence of Kerr-only conservative solitary waves as limiting cases of the dispersion-less dissipative solitons when the spectral filtering becomes an ideal bandpass transmission. By construct, the asymptotic solitary wave shares the properties of an ideal Nyquist pulse, featuring a fully confined ultra-flat spectrum and a transform-limited temporal waveform^24–26^. Owing to their flat and compact spectral support, Nyquist pulses would be instrumental in enabling Kerr frequency combs with unprecedentedly high spectral efficiency, thus benefitting a wide range of applications such as high-capacity telecommunications^27^, spectroscopy^28^, lidar ranging^29^, optical neural computing^30,31^, and radiofrequency photonics^32^.

## Results

### Principle to generate dispersion-less cavity solitons

**Fig. 1:**
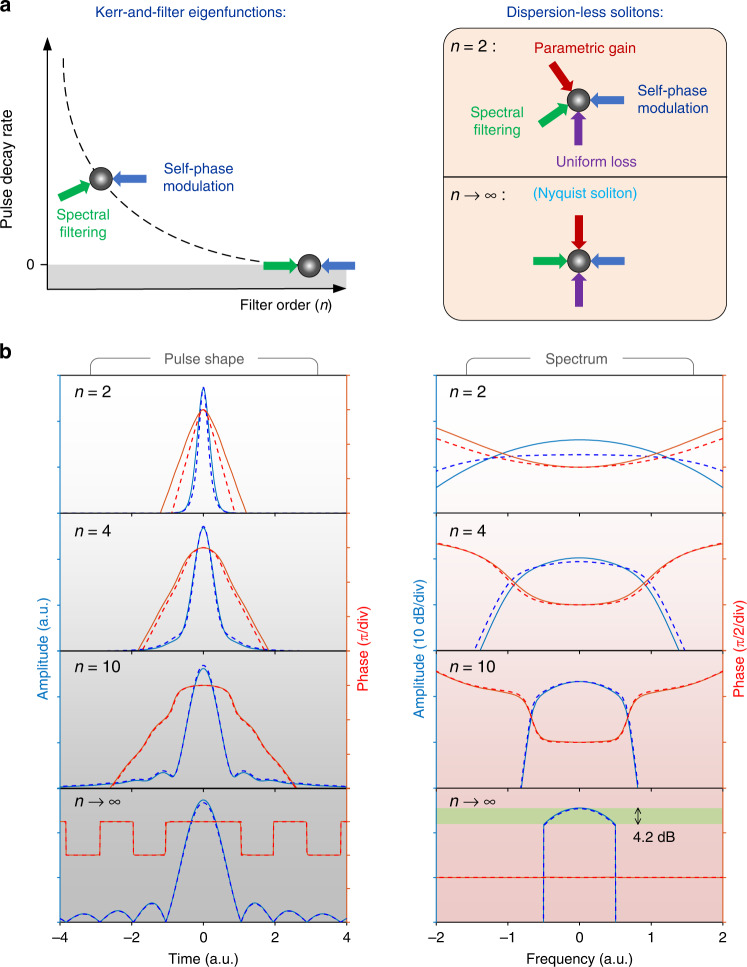
Principle of the dispersion-less cavity soliton. **a** Schematic of the Kerr-and-filter eigenfunctions (left) and the dispersion-less stationary cavity solitons (right). The Kerr-and-filter eigenfunctions have a positive decay rate, which tends to zero when the filter order tends to infinity. Within a coherently pumped cavity, the parametric gain and uniform loss can achieve a composite balance to sustain dispersion-less cavity solitons whatever the filter order. When the filter order $n \to \infty$, a dispersion-less Nyquist soliton results from the double balance between spectral filtering and self-phase modulation on the first hand, and parametric gain and uniform loss on the second hand. **b** Numerical results of the Kerr-and-filter eigenfunctions (dashed lines) and the cavity solitons (solid lines). Left, temporal amplitude and phase; right, spectral amplitude and phase

To search the dispersion-less soliton solutions that form when the average group velocity dispersion becomes negligible, we use the following normalized distributed propagation model (see the Supplementary Section 1.1 for its derivation):

1 $$\frac{{\partial U}}{{\partial Z}} = {{{\mathrm{i}}}}\left| U \right|^2U - \left( {\frac{{{{\mathrm{i}}}}}{\pi }\frac{\partial }{{\partial T}}} \right)^nU - {{{\mathrm{i}}}}\Delta U - U + \left[ {{{{\mathrm{i}}}}C^ \ast U^2 + {{{\mathrm{i}}}}C^2U^ \ast + {{{\mathrm{i}}}}2C\left| U \right|^2} \right] + {{{\mathrm{i}}}}2\left| C \right|^2U$$

where $U$is the field envelope of the optical pulse; $Z$ and $T$ are the scaled spatial and temporal coordinates; $\Delta$ is the pump-cavity phase detuning; $C$ is the homogenous background of the pumped mode in the cavity; $n$ is the filter order. Here, the normalized filter bandwidth out of which the spectral loss exceeds the uniform loss is 1. With the filter order $n \to \infty$, the spectrum of $U$ becomes fully confined in a unit frequency span. The three terms enclosed in square brackets represent the parametric mixing between the soliton and the background pump. The last term is an additional phase shift induced by cross-phase modulation that is usually negligible in comparison to $\Delta$. To gain more insight into the dispersion-less soliton dynamics and how the balance can be achieved between dissipation and Kerr nonlinearity, the following eigenvalue problem is investigated:

2 $${{{\mathrm{i}}}}\left| {U_{{{\mathrm{e}}}}} \right|^2U_{{{\mathrm{e}}}} - \left( {\frac{{{{\mathrm{i}}}}}{\pi }\frac{\partial }{{\partial T}}} \right)^nU_{{{\mathrm{e}}}} = \left( {{{{\mathrm{i}}}}\xi + \lambda } \right)U_{{{\mathrm{e}}}}$$

where $U_{{{\mathrm{e}}}}$ is the eigenfunction of combined SPM and spectral filtering; $\xi$ and $\lambda$ are the imaginary and real parts of the eigenvalue respectively. It turns out that $\lambda$ is always negative: this reflects an overall amplitude loss induced by spectral filtering, whose impact also depends on SPM since the latter is conducive to the broadening of the optical spectrum. Apparently, these eigenfunction solutions are not stationary solutions of the propagation Eq. (1) but represent pulses damped with a decay rate contribution from spectral filtering and SPM that is given by $\left| \lambda \right|$. However, when the pulses are coherently pumped, the energy loss can be compensated by the parametric gain, so that a composite balance can be achieved between all these propagation effects, resulting in the formation of cavity solitons. In the schematic representation of such composite balance by counteracting arrows in Fig. 1a, the vertical dimension represents purely dissipative effects and the horizontal one, purely dispersive contributions. Therefore, the tilt of the parametric gain arrow reflects its frequency dependence, which contributes to the balancing of SPM in absence of chromatic dispersion. More remarkably, we find that for a given value of $\xi$, the energy loss induced by spectral filtering decreases and tends asymptotically to zero with the increase of filter order $n \to \infty$ (see Supplementary Section 1.2). Therefore, in the limit when the filter order tends to infinity, spectral filtering and SPM can balance each other, leading to an eigenfunction that becomes a Kerr-only solitary wave in an ideal bandpass system. In a realistic system, though, we should include linear losses, which can be precisely compensated by the parametric gain.

Figure 1b displays the numerically solved results of Eqs. (1) and (2) with $\xi = \Delta = 20$, at different filter orders *n*. When the filter order increases, the coherently driven pulse described by Eq. (1) gets increasingly closer to the asymptotic soliton described by Eq. (2). Moreover, the soliton spectrum becomes more confined, and the pulse chirp decreases. When the filter order $n \to \infty$, the soliton spectrum becomes fully confined within a unit bandwidth, featuring an intensity variation limited here to 4.2 dB. We also see that the asymptotic soliton adopts a uniform spectral phase, which is perfectly consistent with the fact that, as the losses vanish, the soliton behaves as a stationary conservative soliton. Correspondingly, the soliton temporal waveform becomes a transform-limited Nyquist pulse with characteristic oscillating tails. An approximate closed-form solution can be derived for the Nyquist soliton with an ansatz method. A perturbation analysis of the governing averaged propagation model is performed in the Supplementary Section 1.3, showing that such eigenfunction is a stable attractor for coherently driven Kerr cavities with ideal bandpass transmission and negligible chromatic dispersion.

### Observation of dispersion-less cavity solitons

**Fig. 2:**
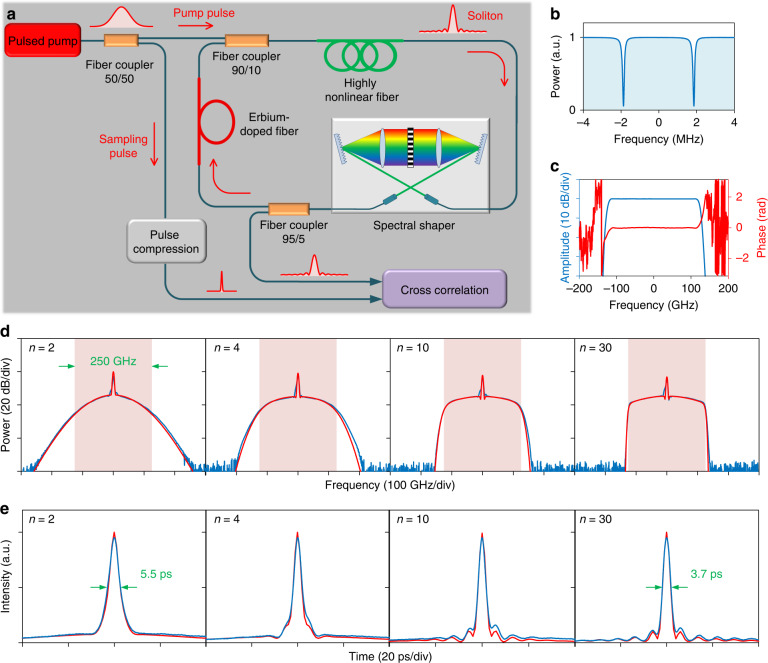
Observation of dispersion-less cavity solitons. **a** Experimental set-up. A spectral shaper is inserted in the cavity for programmable dispersion and filter control. The cavity is synchronously pumped by a sequence of sub-nanosecond Gaussian pulses for soliton generation. **b** Through-port response of the cavity measured with a low-power continuous-wave laser. **c** Roundtrip transfer function of the cavity when the spectral shaper is programmed to a gate bandpass filter and the fiber group velocity dispersion is compensated. **d**, **e** Optical spectrum and temporal intensity waveform of the dispersion-less cavity solitons. Blue, measured; red, simulated. The filter bandwidth is 250 GHz, and the filter order *n* = 2, 4, 10 and 30, respectively. With the increase of filter order, the soliton spectrum becomes more confined in the 250-GHz frequency window (brown region), and the soliton waveform tends to become a Nyquist pulse. The pulse width (FWHM) is 5.5 ps and the estimated chirp parameter is 1 when *n* = 2. The Nyquist soliton when *n* = 30 is nearly chirp-free with a width of 3.7 ps

To study the soliton dynamics, a fiber ring cavity which share the same physical model as miniature microcavities is built. The experimental set-up is shown in Fig. 2a. A spectral shaper is inserted for programmable dispersion and filter control^17,33–35^, and a short length of erbium-doped fiber (EDF) is used to compensate for the roundtrip loss as in ref. ^36^. We note that the incorporation of active EDF here is not intended to build a fiber laser. The EDF length, its optical pumping power, and the pumping strength of the ring cavity are carefully tailored such that the gain provided by the optical amplifier keeps the ring cavity below laser threshold, and that the optical amplifier operates in the linear regime. Figure 2b shows the through-port transmission of the cavity. The total roundtrip length is 54.4 m and the power loss is 16.6%, corresponding to a free spectral range (FSR) of 3.73 MHz and a finesse of 36. Various spectral filtering functions can be implemented by programming the amplitude of the spectral shaper, while the fiber group velocity dispersion is carefully compensated by programming the phase. Figure 2c shows an example of a 250-GHz gate bandpass filter. The spectral shaper has a limited optical resolution of 10 GHz, correspondingly, a maximum filter order of 30 can be achieved.

The roundtrip fiber length is stabilized with respect to the pump laser frequency by using a home-built feedback servo controller. The fiber ring is then synchronously pumped by a sequence of 87.4-ps Gaussian pulses with a peak power of 1.5 W through a 90/10 coupler^37^. The generated soliton is extracted from the 5% output of a 95/5 coupler for ultrafast characterization. To measure the soliton waveform, a high-resolution intensity cross-correlation set-up is built, with the sampling pulses (0.2 ps) generated by spectrally broadening and compressing the pump pulses (more details presented in Supplementary Section 2). Figure 2d, e show the results of the dispersion-less cavity solitons observed when the pump is tuned into the cavity resonance from the blue side (i.e., pump frequency higher than the resonant frequency). The spectral shaper is programmed to apply super-Gaussian filters with orders $n = 2$, 4, 10 and 30, respectively. The filter bandwidth is 250 GHz. An excellent agreement between the experiment and the simulation is obtained. With the increase of the filter order, the soliton spectrum becomes rectangular, and the soliton temporal waveform gets closer to a Nyquist pulse. With the filter order $n = 2$, the measured soliton pulse full width at half maximum (FWHM) is about 5.5 ps while the transform-limited pulse duration assuming a uniform spectral phase would be 3.3 ps, entailing a pulse chirp parameter close to 1. In comparison, the soliton for $n = 30$ is nearly chirp-free with a FWHM of 3.7 ps and becomes close to a Nyquist pulse. For such Nyquist-pulse soliton, about 99.6% of the total spectral power is confined within the 250-GHz frequency window. The latter contains more than 66,000 comb lines within a 6-dB intensity range excluding the pump, corresponding to an unprecedentedly high spectral quality for coherently driven Kerr frequency combs^6^.

### Nyquist soliton transition dynamics and its existence domain

**Fig. 3:**
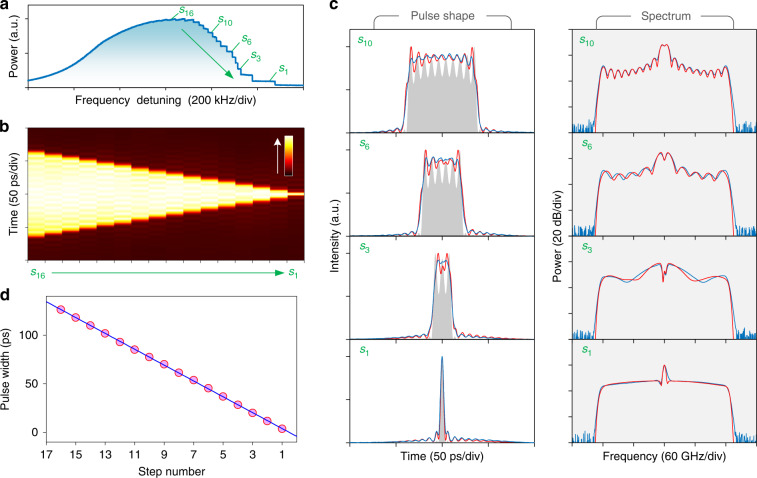
Nyquist soliton transition dynamics under pulsed pumping. **a** Intracavity power versus pump frequency detuning. Steps corresponding to soliton state transitions are clearly observed. **b** Evolution of the soliton waveform with the transition step. The color gradient represents the optical intensity. **c** Pulse shape (left) and spectrum (right) at different steps marked in (**a**). Blue, measured; red, simulated. The gray shadows indicate the simulated soliton molecules sustained by a balance between Kerr nonlinearity and spectral filtering alone. With the increase of pump detuning, the soliton number decreases one by one, until a single Nyquist soliton is formed at the last step. **d** Total width of the soliton molecule at each step. Red circles, measured; blue line, linear fit

Using the maximum filter order $n = 30$, we find that the Nyquist soliton can be generated deterministically under pulsed pumping. Figure 3a shows the mean intracavity power trace when the pump laser scans across the resonance from the blue side. Steps corresponding to soliton state transitions can be clearly observed. In contrast to conventional dispersion-enabled cavity solitons which are usually excited stochastically through chaotic modulational instability^23^, the Nyquist soliton generation is rather deterministic with an absence of noisy region observed from the intracavity power trace. The evolution of the soliton waveform with the transition step is visualized in Fig. 3b, with some selected states and their spectra detailed in Fig. 3c (see Supplementary Section 2.3 for the full data). All the solitary structures can be stably maintained when the scanning laser stops at the corresponding steps. The solitons appear as square pulses with characteristic oscillating peaks and spectral sidelobes. The pulse duration is quantized and has a very good linear relation with the power step number (Fig. 3d). After each transition step, the pulse width decreases by about 8.1 ps. Numerical simulations reveal that these square pulses are associated with compact Nyquist soliton molecules (i.e., closely bound solitons^5,38^) which can also be sustained in ideal bandpass systems by the balance between Kerr nonlinearity and spectral filtering alone (Supplementary Section 1.4). With the increase of pump frequency detuning, the soliton number in the molecule decreases one by one and a single Nyquist soliton is ultimately formed. It is found that the soliton transition process can be affected by the desynchronization between the pump pulse repetition rate and the cavity FSR (Supplementary Section 2.4). In some cases, the existence region of some intermediate states may be too narrow to be observed and the soliton number decreases by two or more in a single transition step.

**Fig. 4:**
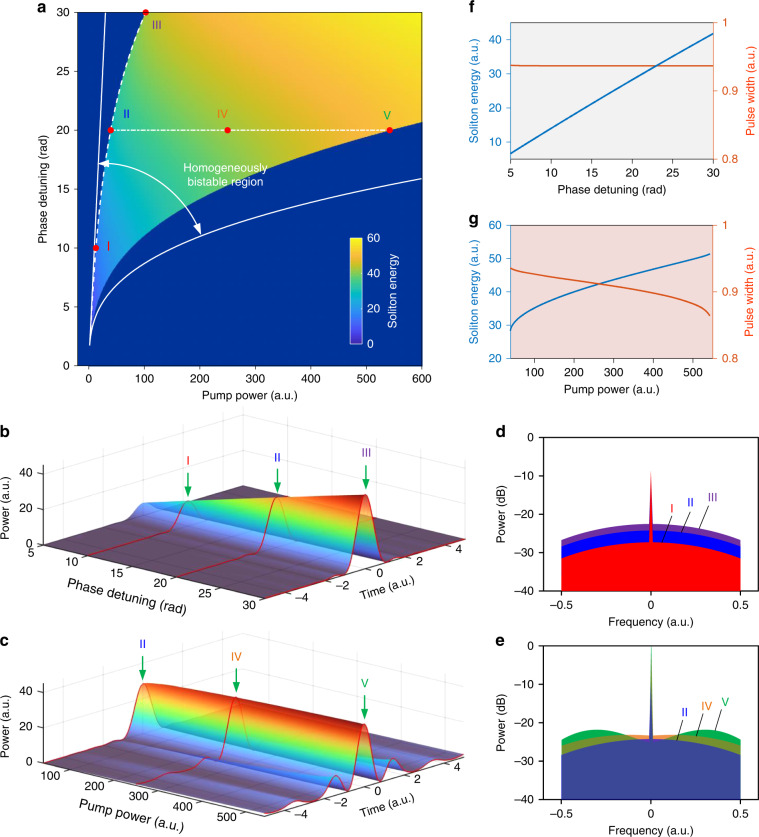
Evolution of Nyquist solitons. **a** Soliton existence region. The dispersion-less Nyquist soliton can be maintained in a wide parameter space within the homogenously bistable region. **b**, **c** Evolution of the pulse shape along the dash and dash-dotted lines in (**a**)**. d**, **e** Soliton spectra at different locations marked in (**a**). For I, II, and III, the pump phase detuning is 10, 20, and 30, respectively. For II, IV, and V, the pump phase detuning is 20 and the pump power is 40, 250, and 540, respectively. **f**, **g** Plots of the pulse energy and width along the dash and dash-dotted lines in (**a**)

To explore the existence region of the single Nyquist soliton, a parameter scanning is performed numerically based on the normalized continuously driven equation. The results are shown in Fig. 4a. The Nyquist soliton can be maintained in a relatively wide parameter space that lies within the homogenously bistable region. Figure 4b, c illustrate the evolution of the soliton pulse shape with the pump phase detuning and the pump power, respectively. The soliton spectra at some selected locations marked in Fig. 4a are plotted in Figs. 4d and 4e. Along the lower boundary of the pump power (the minimum pump power required to maintain the soliton), the soliton intensity increases linearly with the increase of pump detuning. Meanwhile, the pulse width keeps nearly unchanged since the spectral bandwidth remains fully confined by the gate filter in the frequency domain. Detailed plots of the pulse energy and width are shown in Fig. 4f, g. The energy growing slope is about 1.5 and the pulse width is 0.94. This indicates that the ideal Nyquist soliton is free from energy-width scaling, in sharp contrast to the conventional NLSE solitons enabled by the second-order dispersion for which the energy scales with the inverse of the pulse duration. Therefore, the Nyquist soliton can, in theory, be promoted indefinitely while keeping almost the same pulse and spectral shape (more discussions about the scaling law of dispersion-less solitons are presented in Supplementary Section 1.2.2). When the pump detuning is kept constant, the soliton energy also increases slowly with the pump power, with an average slope of 0.035. A plot of the overlaid soliton pulses under different pump power levels is shown in Supplementary Section 2.5. As the pump power increases, the main pulse width keeps nearly unchanged while the oscillating tails get more prominent. In the frequency domain, the spectral components close to the passband edge get more enhanced, giving rise to an increasingly flat spectrum. Along the dash-dotted line in Fig. 4a for which the pump phase detuning is kept at 20, the best achievable spectral flatness is about 2.3 dB when the pump power varies between the lower and upper boundaries. The change of the soliton pulse shape under increasing pump power levels can be understood intuitively as follows. When the pump power is high, for a pulse shape primarily determined by the balance between spectral filtering and SPM (i.e., Kerr-and-filter eigenfunctions following Eq. (2)), the parametric gain will be obviously larger than that required for compensating for the uniform loss. The soliton shape thus changes adaptively such that a new composite balance is achieved between the physical effects involved. A schematic illustration is shown in Supplementary Fig. S10b. The extended parametric gain arrow and its tilt indicate the difference in the balanced state compared to when the pump power is low. Due to the difficulty of expressing the distorted soliton pulse in closed form, the analytical description of this phenomenon is a challenging task that we leave for future work.

### Relation between filter-driven and dispersion-driven solitons

**Fig. 5:**
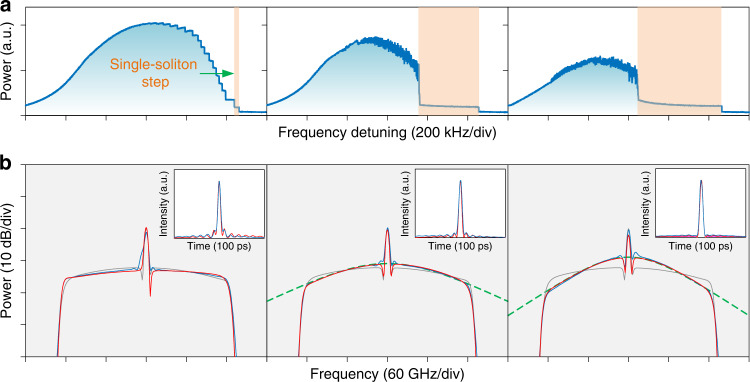
Relation between filter-driven and dispersion-driven solitons. **a** Intracavity power versus pump frequency detuning. From left to right, the net roundtrip dispersion is 0.63, −1.06, and −4.22 ps^2^, respectively. A chaotic region with higher noise can be observed when the dispersion is negative (anomalous). **b** Soliton spectrum and pulse shape (inset) at the corresponding single-soliton steps (brown regions) indicated in (**a**). Blue, measured; red, simulated. The result with no dispersion is also shown for comparison (gray solid line). With the increase of anomalous dispersion, the pulse transitions from a filter-driven Nyquist soliton to a conventional dispersion-driven nonlinear Schrödinger soliton for which the spectral envelope can be well fitted by a hyperbolic secant function (green dash line)

When the net group velocity dispersion is not zero in the fiber loop, both the dispersion and the spectral filter will be responsible for soliton pulse shaping. Figure 5 shows the results when the spectral shaper is programmed to a 250-GHz gate filter and different amounts of second-order dispersion are applied. The soliton spectrum becomes slightly flatter when the net roundtrip dispersion is slightly normal (0.63 ps^2^). However, the soliton existence region becomes narrower, and it is more difficult to get stable single Nyquist soliton when the dispersion is further increased. When the net dispersion is anomalous, the spectral intensity around the pump wavelength gets more pronounced. Two cases with net dispersion values of −1.06 and −4.22 ps^2^ are shown. With an increase of the net anomalous dispersion, the soliton tends to adopt hyperbolic-secant spectrum that is typical from conventional dispersion-driven solitons. The oscillating tails aside the main pulse, which are a signature of a Nyquist pulse, get diminished. A chaotic region with higher noise can also be observed from the intracavity power transition trace, which is attributed to the modulational instability in the anomalous dispersion region^23^. For all the three cases shown in Fig. 5b, the solitons are very close to transform-limited pulses with no obvious chirp.

## Discussion

In summary, this article demonstrates the existence of dispersion-less cavity solitons sustained by the balance between dissipation and Kerr nonlinearity. In addition to deepening the dissipative soliton paradigm, our study reveals an unexpected connection between dissipative and conservative cavity solitons by virtue of Nyquist pulses. We have developed a theoretical model relating the solitons to the eigenfunctions of SPM and spectral filtering effects. When the filter order tends to infinity, a Nyquist-pulse-like soliton is demonstrated. Such Nyquist soliton possesses several outstanding merits, including a fully confined flat spectrum, a transform-limited pulse shape, deterministic generation under pulsed pumping, and immunity from energy-width scaling.

We note that the dispersion-less solitons presented here are distinct from the “zero-dispersion” solitons that have been recently demonstrated in driven passive cavities^14,15^, photonic crystal waveguides^16^ and mode-locked lasers^17^ when the average second-order dispersion is vanishing. Indeed, the formation of solitons in refs. ^14–17^ is supported by the interaction involving the higher-order group velocity dispersion terms along with the Kerr nonlinearity (thus is still dispersion-driven). By “dispersion-less” in this article, we emphasize that the soliton is primarily stabilized by the balance between SPM and spectral filtering. The participation of any order of dispersion is not a necessitate. Even when there is a weak dispersion, it will just act as a higher-order perturbation. Such a soliton formation mechanism has never been revealed in coherently driven optical cavities, to the best of our knowledge. Moreover, whereas our experimental fiber ring is constructed from discrete elements with alternating normal and anomalous dispersion, the soliton dynamics is fundamentally different from the stretched-pulse mode-locking^10,11^. Such statement is supported by a comparison between the soliton spectra measured before and after the spectral shaper in our set-up, see the Supplementary Section 2.6, showing no significant difference. This is explained by the fact that the local dispersion length is always much larger than the cavity length for the pulse duration involved, justifying the prevalence of the path-averaged cavity dynamics.

We have experimentally generated dispersion-less solitons having a pulse width in the range of a few picoseconds, which is typical of coherently driven temporal solitons obtained within fiber resonators^22,36^. For some applications, shorter pulses in the femtosecond range featuring a larger frequency comb bandwidth would be required. Although the spectral shaper we employ is capable of compensating for the fiber loop dispersion over a large bandwidth^39^, stable femtosecond soliton generation becomes very challenging in our experiments due to the increased requirement of a precise synchronization between the pump pulse and the cavity soliton. Furthermore, when the local dispersion length of femtosecond solitons becomes comparable to or shorter than the fiber length of each section, the soliton evolution departs from the dispersion-less case and eventually transition to the conventional stretched-pulse mode-locking regime. It is therefore quite impractical to generate femtosecond dispersion-less solitons with the present set-up. The purpose of our work is to demonstrate the concept of dispersion-less dissipative solitons while benefitting from the versatile fiber ring cavity architecture, which can be subsequently scaled to femtosecond broadband solitons by using other photonic platforms. For instance, a fiber-based viable solution can be envisaged by using miniature Fabry–Pérot fiber resonators fabricated with fiber end polishing and high-reflection coatings^37^. By choosing a proper fiber with close-to-zero dispersion at the pump wavelength and with bandpass coating, a spectrally confined fiber resonator with nearly vanishing dispersion can be obtained to support dispersion-less solitons. Another very promising implementation for practical applications is to generate femtosecond dispersion-less solitons within on-chip integrated microresonators. Flexible dispersion engineering and filter control can be achieved by tailoring the waveguide dimension and employing photonic crystal structures^40,41^. The micro-ring and Fabry–Pérot conceptual designs are illustrated in the Supplementary Section 2.7. Numerical simulations are performed by considering the typical parameters of the silicon nitride platform: they show that femtosecond dispersion-less solitons having a flat comb spectrum spanning over 100 nm are accessible. Flat microcombs are very attractive for various applications such as high-speed communications, lidar ranging and optical computing.

Finally, in our dispersion-less soliton experiments, we do not observe the Gordon–Kelly resonant sidebands that cause instabilities of high-energy pulses in dispersion-driven soliton mode-locked fiber lasers^17,42^. Since the group velocity dispersion is absent, the formation mechanism of Gordon–Kelly sidebands, namely constructive interference between the soliton and the dispersive wave, is no longer applicable. This implies an improved robustness of the dispersion-less solitons compared to the conventional dispersion-driven solitons. The discovery of dispersion-less dissipative solitons will be instrumental in the development of novel ultrafast lasers and frequency combs with previously unattainable spectral and temporal features.

## Materials and methods

### Soliton generation and characterization

The fiber ring cavity is mainly composed of a 21-m highly nonlinear fiber (YOFC NL-1550-NEG) and a ~30-m standard single-mode fiber. A length of 0.6-m erbium-doped fiber (EDF, YOFC EDF13) is used to compensate for the loss. The EDF is pumped by a 1.8-W 976-nm laser, offering a gain of ~4.1 dB. The total cavity length is about 54.4 m which is typical for coherently driven fiber solitons^22,36^. The long fiber cavity facilitates the experiments by boosting the Kerr nonlinearity per roundtrip and reducing the required pump power level.

A commercial spectral shaper (Finisar WaveShaper 1000A) is inserted for programmable dispersion and filter control. Its dispersion trimming capability is characterized by a dispersion-bandwidth product of 80 ps which is sufficient for our experiments^39^. To perform an accurate dispersion compensation, a transform-limited pulse train (FWHM: ~0.5 ps) from a mode-locked fiber laser is sent through the fiber link when the loop is open. The output pulse shape is monitored through intensity autocorrelation. The phase of the spectral shaper is then adjusted iteratively to make the output pulse transform-limited again. The cavity roundtrip transfer function after dispersion compensation is further checked with a commercial optical vector network analyzer (Luna) and the results are shown in Fig. 2c. The pulsed pump is generated by modulating a continuous-wave narrow-linewidth laser (OEwaves) with the signals from a pulse pattern generator (Anritsu), and is amplified to a peak power of 1.5 W for soliton sustainment. The amplified spontaneous emission noise is rejected by a narrow-band filter. To generate solitons, the pump laser frequency is manually tuned into the cavity resonance from the higher-frequency side. In the meanwhile, the relative detuning is stabilized by a home-built feedback servo controller to mitigate environmental interference. The soliton spectrum is measured by a commercial spectrum analyzer (Yokogawa) with a resolution of 0.02 nm; and the waveform is measured by a home-built intensity cross correlator having a resolution of 0.2 ps.

### Theoretical model and numerical simulations

The results in Fig. 1b are obtained by numerically finding the solutions of Eq. (1) (with the left side set to zero) and (2) with the Newton–Rapson method. The phase shifting parameters are $\xi = \Delta = 20$. Note that Eq. (1) is identical to the following equation

3 $$\frac{{\partial \psi }}{{\partial Z}} = - \left( {1 + {{{\mathrm{i}}}}\Delta } \right)\psi + {{{\mathrm{i}}}}\left| \psi \right|^2\psi - \left( {\frac{{{{\mathrm{i}}}}}{\pi }\frac{\partial }{{\partial T}}} \right)^n\psi + S$$

where $\psi \left( {Z,T} \right) = U\left( {Z,T} \right) + C$ the total intracavity field; and $S$ is the pump field. In each case, the pump power $\left| S \right|^2$is adjusted near its minimum value that is required to maintain the stable soliton. For the filter order $n = 2$, 4, 10 and $\infty$, the pump power $\left| S \right|^2 = 420$, 280, 120 and 25, respectively. The homogenous background $C$ is calculated by finding the lower-branch solution of Eq. (3) with the derivative terms set to zero.

When solving the eigenfunctions of Eq. (2), the real part of the eigenvalue (i.e., $\lambda$) can also be obtained at the same time as an unknown variable that depends on $\xi$.

The parameter scanning results in Fig. 4 are obtained by numerically integrating Eq. (3) with the standard split-step Fourier method until the intracavity field reaches a stable state. The cavity roundtrip time is 100. Note that the plots of Figs. 4d and 4e include both the soliton and the homogenous pump background, different from Fig. 1b which shows only the soliton.

The simulation results in Figs. 2, 3 and 5 for the fiber cavity are obtained with the split-step Fourier method, based on a simplified model in which the effects of Kerr nonlinearity, dispersion and loss are considered separately by lumped terms in one roundtrip. The field at the end of the (*m* + 1)-th round is related to that after the *m*-th round by

4 $$A_{m + 1} = \sqrt {\left( {1 - \theta } \right)\left( {1 - \alpha _L} \right)} e^{ - {{{\mathrm{i}}}}\left( {\delta _L + \gamma P_mL} \right)}\widehat FA_m + \sqrt \theta A_{{{\mathrm{p}}}}$$

where $A$ is the field amplitude normalized such that $P = \left| A \right|^2$ represents the field power; $L$ is the roundtrip length; $\alpha _L$ is the universal roundtrip power loss for all the modes; $\delta _L$ is the pump-cavity phase detuning; $\gamma$ is the average Kerr coefficient; $\theta$ is the pump-cavity power coupling ratio; and $A_{{{\mathrm{p}}}}$ is the pump field. The operator $\widehat F$ accounts for the effects that can be easily applied in the frequency domain, including the filtering loss, the group velocity dispersion and the pump-cavity desynchronization (see Eq. (S28) in Supplementary Section 1.5 for more details). The simulation parameters are $\theta = 10.4\%$, $\alpha _L = 16.6\%$, $\gamma = 4.6 \times 10^{ - 3}{{{\mathrm{ m}}}}^{ - 1}{{{\mathrm{W}}}}^{ - 1}$, and $L = 54.6{{{\mathrm{ m}}}}$. The pump pulse is Gaussian with a FWHM of 87.4 ps and a peak power of 1.5 W. The power transfer function of the super-Gaussian filter implemented by the spectral shaper is given by

5 $$\left| {H\left( \nu \right)} \right|^2 = e^{ - \left[ {2\left( {\nu - \nu _0} \right)/B} \right]^n}$$

where $\nu _0$, $B$, and $n$ represent the filter central frequency, bandwidth, and order, respectively.

## Supplementary information

Supplementary material

## References

[1] Russell, S. J. *Report on Waves: Made to the Meetings of the British Association in 1842–1843* (Forgotten Books, 1845).

[2] Boardman, A. D. & Sukhorukov, A. P. *Soliton-Driven Photonics* (Springer, 2001).

[3] Akhmediev, N., Ankiewicz, A. & Akhmediev, N. *Dissipative Solitons: From Optics to Biology and Medicine* (Springer, 2008).

[4] Haus HA, Wong WS (1996). Solitons in optical communications. Rev. Mod. Phys..

[5] Grelu P, Akhmediev N (2012). Dissipative solitons for mode-locked lasers. Nat. Photonics.

[6] Kippenberg TJ (2018). Dissipative Kerr solitons in optical microresonators. Science.

[7] Chong A (2006). All-normal-dispersion femtosecond fiber laser. Opt. Express.

[8] Huang SW (2015). Mode-locked ultrashort pulse generation from on-chip normal dispersion microresonators. Phys. Rev. Lett..

[9] Spiess C (2021). Chirped dissipative solitons in driven optical resonators. Optica.

[10] Haus HA (1995). Stretched-pulse additive pulse mode-locking in fiber ring lasers: theory and experiment. IEEE J. Quantum Electron..

[11] Dong X (2020). Stretched-pulse soliton Kerr resonators. Phys. Rev. Lett..

[12] Renninger WH, Chong A, Wise FW (2011). Amplifier similaritons in a dispersion-mapped fiber laser. Opt. Express.

[13] Parra-Rivas P, Gomila D, Gelens L (2017). Coexistence of stable dark- and bright-soliton Kerr combs in normal-dispersion resonators. Phys. Rev. A.

[14] Li ZD (2020). Experimental observations of bright dissipative cavity solitons and their collapsed snaking in a Kerr resonator with normal dispersion driving. Optica.

[15] Anderson, M. H. et al. Zero-dispersion Kerr solitons in optical microresonators. *Nat. Commun*. **13**, 4764 (2020). 10.1038/s41467-022-31916-x PMC9376110 35963859

[16] Blanco-Redondo A (2016). Pure-quartic solitons. Nat. Commun..

[17] Runge AFJ (2020). The pure-quartic soliton laser. Nat. Photonics.

[18] Turitsyn SK (2016). Dissipative solitons in fiber lasers. Phys. Uspekhi.

[19] Grelu, P. in *Handbook of Laser Technology and Applications**(Laser Design & Laser Systems)* 2nd edn, Vol. II (eds Guo, C. L. & Singh, S. C.) Ch. 39 (Taylor & Francis, 2021).

[20] Haelterman M, Trillo S, Wabnitz S (1992). Dissipative modulation instability in a nonlinear dispersive ring cavity. Opt. Commun..

[21] Wabnitz S (1993). Suppression of interactions in a phase-locked soliton optical memory. Opt. Lett..

[22] Leo F (2010). Temporal cavity solitons in one-dimensional Kerr media as bits in an all-optical buffer. Nat. Photonics.

[23] Herr T (2014). Temporal solitons in optical microresonators. Nat. Photonics.

[24] Nakazawa M, Yoshida M, Hirooka T (2014). The Nyquist laser. Optica.

[25] Turitsyn SK, Bogdanov S, Redyuk A (2020). Soliton-sinc optical pulses. Opt. Lett..

[26] Nakazawa M (2012). Ultrahigh-speed ‘orthogonal’ TDM transmission with an optical Nyquist pulse train. Opt. Express.

[27] Marin-Palomo P (2017). Microresonator-based solitons for massively parallel coherent optical communications. Nature.

[28] Suh MG (2016). Microresonator soliton dual-comb spectroscopy. Science.

[29] Riemensberger J (2020). Massively parallel coherent laser ranging using a soliton microcomb. Nature.

[30] Feldmann J (2021). Parallel convolutional processing using an integrated photonic tensor core. Nature.

[31] Xu XY (2021). 11 TOPS photonic convolutional accelerator for optical neural networks. Nature.

[32] Wu JY (2018). RF photonics: an optical microcombs’ perspective. IEEE J. Sel. Top. Quantum Electron..

[33] Weiner AM (2000). Femtosecond pulse shaping using spatial light modulators. Rev. Sci. Instrum..

[34] Schröder J (2010). Dark and bright pulse passive mode-locked laser with in-cavity pulse-shaper. Opt. Express.

[35] Peng JS, Boscolo S (2016). Filter-based dispersion-managed versatile ultrafast fibre laser. Sci. Rep..

[36] Englebert N (2021). Temporal solitons in a coherently driven active resonator. Nat. Photonics.

[37] Obrzud E, Lecomte S, Herr T (2017). Temporal solitons in microresonators driven by optical pulses. Nat. Photonics.

[38] Stratmann M, Pagel T, Mitschke F (2005). Experimental observation of temporal soliton molecules. Phys. Rev. Lett..

[39] Application note: Dispersion trimming using the programming group delay capability of the Waveshaper family of programmable optical processors. https://ii-vi.com/dispersion-trimming (2022).

[40] Yu SP (2019). Photonic-crystal-reflector nanoresonators for Kerr-frequency combs. ACS Photonics.

[41] Lucas, E. et al. Inverse spectral design of Kerr microcomb pulses. In *Proc. SPIE 11672, Laser Resonators, Microresonators, and Beam Control XXIII* 1167205 (SPIE, 2021).

[42] Kelly SMJ (1992). Characteristic sideband instability of periodically amplified average soliton. Electron. Lett..

