# Supplementary material for: Dispersion-less Kerr solitons in spectrally confined optical cavities
Source: Light Sci Appl. 2023 Jan 9;12:19. doi: 10.1038/s41377-022-01052-8 (PMC9826788; doi:10.1038/s41377-022-01052-8)
Supplement: Supplementary file 1 — Supplementary material [file 41377_2022_1052_MOESM1_ESM.pdf]

Supplementary Materials for

Dispersion-less Kerr solitons in spectrally confined optical cavities

Xiaoxiao Xue[1*], Philippe Grelu[2], Bofan Yang[1], Mian Wang[1], Shangyuan Li[1], Xiaoping Zheng[1#], and Bingkun Zhou[1]

[1]*Department of Electronic Engineering, Beijing National Research Center for Information Science and Technology, Tsinghua University, Beijing 100084, China.*
[2]*Laboratoire ICB UMR 6303 CNRS, Université Bourgogne Franche-Comté, 21000 Dijon, France.*
*xuexx@tsinghua.edu.cn; [#]xpzheng@tsinghua.edu.cn

1. Theoretical model

1.1 Mean-field equation for dispersion-less cavity solitons

When only the n-th order spectral loss is considered, the field evolution can be described by the following mean-field equation[S1-S3]

$$\frac{\partial A}{\partial z} = -(\alpha + \mathrm{i}\delta)A + \mathrm{i}\gamma|A|^2 A - \rho\left(\mathrm{i}\frac{\partial}{\partial t}\right)^n A + \kappa A_\mathrm{p}, \tag{S1}$$

where A is the field envelop; t is the time; z is the propagation distance; α is the uniform loss; δ is the pump-cavity phase detuning; γ is the Kerr nonlinear coefficient; ρ is the spectral loss coefficient; A_p is the pump field; and κ is the pump coupling ratio. Here we only consider the case when n is even. The spectral loss per unit length is $\rho\omega^n$ where ω is the angular frequency. The amplitude transfer function of the spectral filtering effect related to a propagation distance of L is then $e^{-\rho\omega^n L}$, i.e., a super-Gaussian function.

The normalized form of Eq. (S1) is given by

$$\frac{\partial \psi}{\partial Z} = -(1 + \mathrm{i}\Delta)\psi + \mathrm{i}|\psi|^2\psi - \left(\frac{\mathrm{i}}{\pi}\frac{\partial}{\partial T}\right)^n \psi + S, \tag{S2}$$

with

$$Z = \alpha z,\ T = Bt,\ \psi = AB\sqrt{\frac{\gamma}{\alpha}},\ \Delta = \frac{\delta}{\alpha},\ S = \frac{\kappa B A_\mathrm{p}}{\alpha}\sqrt{\frac{\gamma}{\alpha}},\ B = \frac{1}{\pi}\left(\frac{\alpha}{\rho}\right)^{1/n}. \tag{S3}$$

Suppose the solution is a soliton (U) sitting atop of a homogenous background (C)

$$\psi(Z,T) = U(Z,T) + C\,. \tag{S4}$$

Substituting Eq. (S4) into Eq. (S2), we get the equations for U and C respectively as follows

$$-(1+\mathrm{i}\Delta)C + \mathrm{i}|C|^2 C + S = 0\,, \tag{S5}$$

$$\frac{\partial U}{\partial Z} = \mathrm{i}|U|^2 U - \left(\frac{\mathrm{i}}{\pi}\frac{\partial}{\partial T}\right)^n U - \mathrm{i}\Delta U - U + \left[\mathrm{i}C^* U^2 + \mathrm{i}C^2 U^* + \mathrm{i}2C|U|^2\right] + \mathrm{i}2|C|^2 U\,. \tag{S6}$$

For bright solitons, C is the solution on the lower branch of the bi-stability curve[S4]. When searching for stationary solitons, solving Eqs. (S5) and (S6) in sequence is intrinsically equivalent to solving Eq. (S2).

1.2 Eigen functions of spectral filtering and self-phase modulation

1.2.1 Evolution with filter order

The eigenfunctions of combined spectral filtering and self-phase modulation (SPM) obey

$$\mathrm{i}|U_{\mathrm{e}}|^2 U_{\mathrm{e}} - \left(\frac{\mathrm{i}}{\pi}\frac{\partial}{\partial T}\right)^n U_{\mathrm{e}} = (\mathrm{i}\xi + \lambda)U_{\mathrm{e}}\,, \tag{S7}$$

where λ and ξ represent the real and imaginary parts of the eigenvalue respectively. It is noted that λ and ξ are correlated. For a given ξ, the eigenfunction U_{e} and the real eigenvalue λ can be obtained by numerically solving Eq. (S7) with the Newton-Rapson method. Figure S1 shows the evolution of U_{e} and λ with the filter order when $\xi = 1$. It is found that λ is always negative, representing an overall amplitude loss induced by SPM in combination with spectral filtering. Remarkably, $|\lambda|$ decreases with the increase of filter order n. When the filter order $n \to \infty$, we have $|\lambda| \to 0$.

Although the eigenfunctions do not necessarily constitute stable solitons, they provide important insights for the dispersion-less dissipative solitons (as is shown by the comparison in Fig. 1**b** of the main paper) and may be regarded as a kind of soliton "kernel". One particularly interesting case is when $|\lambda| \to 0$ with $n \to \infty$. Note that the spectral loss can be expressed in the frequency domain as

$$H(\nu) = (2\nu)^n \tag{S8}$$

where ν is the optical frequency. Therefore, when $n \to \infty$, it turns to an ideal gate filter with a unit bandwidth

$$H(\nu) = \begin{cases} 0, & -1/2 \le \nu \le 1/2 \\ \infty, & \text{others} \end{cases}. \tag{S9}$$

The vanishment of $|\lambda|$ implies that in this limiting case, an energy-conserved balance can be achieved between SPM and the gate bandpass filtering. The resulting waveform is close to a Nyquist pulse with a fully confined spectrum.

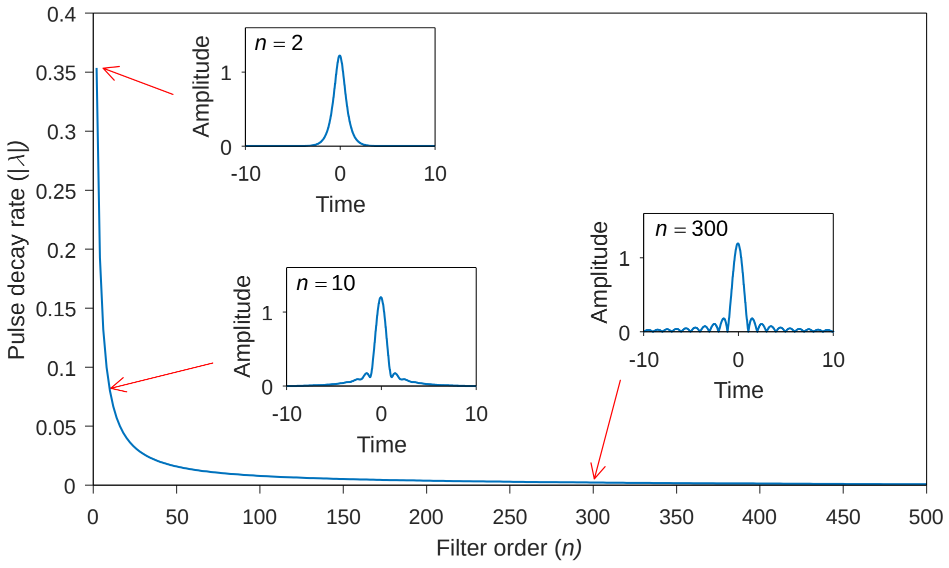

Fig. S1. Evolution of pulse shape and decay rate with the filter order. The imaginary eigenvalue $\xi = 1$.

1.2.2 Scaling law

The scaling law for the eigenfunctions can be easily checked as

$$\xi \to \eta\xi,\ \lambda \to \eta\lambda,\ U_e \to \eta^{1/2}U_e,\ T \to T\eta^{1/n}, \tag{S10}$$

where η is a positive number. The pulse energy is then

$$E = \int \eta \left|U_e\left(\eta^{1/n}T\right)\right|^2 \mathrm{d}T = \eta^{1-1/n}E_0, \tag{S11}$$

where E_0 is the energy of $U_e(T)$, given by

$$E_0 = \int \left| U_{\mathrm{e}}(T) \right|^2 \mathrm{d}T \,. \tag{S12}$$

And the pulse width is

$$W = \eta^{-1/n} W_0 \,, \tag{S13}$$

where W_0 is the pulse width of $U_{\mathrm{e}}(T)$. The relation between E and W is thus given by

$$E = E_0 W_0^{n-1} \left(\frac{1}{W} \right)^{n-1} = C_n \left(\frac{1}{W} \right)^{n-1} \,, \tag{S14}$$

where $C_n = E_0 W_0^{n-1}$ is a constant depending on the filter order. The pulse energy scales with the $(n-1)$-th power of the inverse pulse duration. Again, the interesting condition is when $n \to \infty$. The pulse width will be nearly unchanged when the pulse energy increases (also see Eq. (S10)), as a result of a spectrum that is fully confined by an ideal gate filter.

1.2.3 Nyquist-pulse-like solution

Equation (S7) is not integrable, thus no precise analytic solution can be obtained for U_{e}. In the next, an approximate solution is constructed for the Nyquist-pulse-like eigenfunction with $n \to \infty$. Intuitively, we assume the spectral envelop when $\xi = 1$ is given by

$$\mathbb{U}_{\mathrm{e}} = \mathcal{F}\{U_{\mathrm{e}}\} = a \left[\frac{1 + \cos(b\nu)}{2} \right] \,, \tag{S15}$$

where $\mathcal{F}$ represents Fourier transform, a and b are constants. Transforming Eq. (S7) with $\xi = 1$ and $n \to \infty$ to the frequency domain yields

$$\mathrm{i} \left(\mathbb{U}_{\mathrm{e}} \otimes \mathbb{U}_{\mathrm{e}}^{*} \right) \otimes \mathbb{U}_{\mathrm{e}} - H \mathbb{U}_{\mathrm{e}} = \mathrm{i} \mathbb{U}_{\mathrm{e}} \,, \tag{S16}$$

where H is the ideal gate filter (i.e., Eq. (S9)). The values of a and b may be found analytically by using the variational method. A much simpler and likely more accurate approach is just fitting the numerical results with Eq. (S15). The retrieved parameters are $a = 1.382$ and $b = 2.669$. The time-domain pulse shape is then calculated by

inverse Fourier transform as follows

$$U_{\mathrm{e}} = \mathcal{F}^{-1}\{\mathcal{U}_{\mathrm{e}}\} = \frac{a\sin(\pi T)}{\pi T} + \frac{2ab\sin\left(\frac{b}{2}\right)\cos(\pi T) - 4\pi a\cos\left(\frac{b}{2}\right)T\sin(\pi T)}{b^2 - 4\pi^2 T^2}. \tag{S17}$$

Figure S2 compares the numerical results with the calculated results based on Eqs. (S15) and (S17), showing very good agreement. According to the scaling law given by Eq. (S10), the eigenfunction spectrum for arbitrary ξ can be written as

$$\mathcal{U}_{\mathrm{e}} = \sqrt{\xi} a\left[\frac{1+\cos(b\nu)}{2}\right] . \tag{S18}$$

The results above are obtained with the normalized equation. For any practical experimental configuration, the soliton parameters can be easily obtained by performing denormalization.

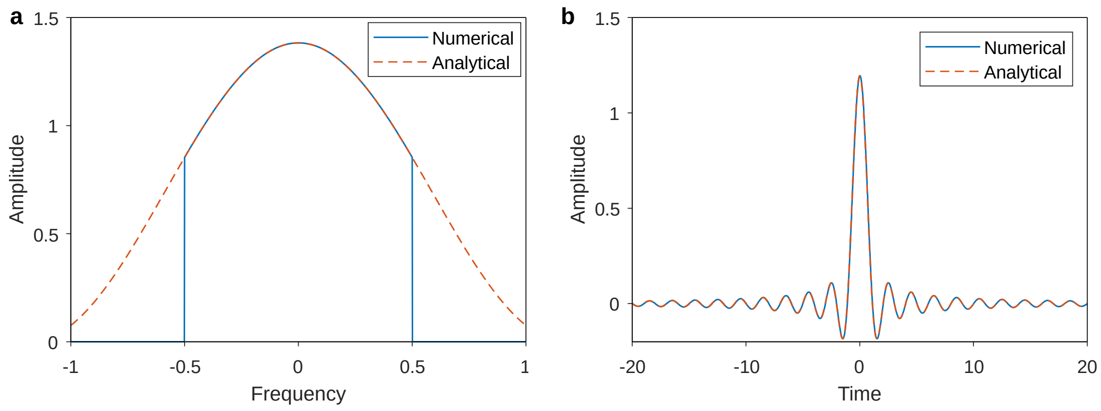

Fig. S2. Comparison between numerical and approximate analytical results for the Nyquist-pulse-like eigenfunction. a Spectrum. **b** Pulse shape.

1.3 Nyquist cavity solitons

In the next, based on Eq. (S15), we derive an approximate solution for the Nyquist-pulse-like solitons in coherently driven cavities. The procedure is similar to perturbation analysis for conventional dispersion-driven solitons[S4], but performed in the frequency domain because the time-domain pulse shape of Eq. (S17) is complicated. We first transform Eq. (S6) to the frequency domain as follows

$$\frac{\partial \mathbb{U}}{\partial Z}=\mathrm{i}\left(\mathbb{U}\otimes\mathbb{U}^{*}\right)\otimes U-H\mathbb{U}-\mathrm{i}\Delta\mathbb{U}-\mathbb{U}+\left[\mathrm{i}C^{*}\mathbb{U}\otimes\mathbb{U}+\mathrm{i}C^{2}\mathbb{U}^{*}+\mathrm{i}2C\mathbb{U}\otimes\mathbb{U}^{*}\right]+\mathrm{i}2\left|C\right|^{2}U \tag{S19}$$

Suppose the soliton spectrum can be written as

$$\mathbb{U}=\eta\left[\frac{1+\cos\left(b\nu\right)}{2}\right]e^{\mathrm{i}\phi}=\eta Q e^{\mathrm{i}\phi} \quad \text{with } -1/2<\nu<1/2, \tag{S20}$$

where η and ϕ are spectral amplitude and phase respectively. For convenience, we use the simplified denotation $Q=\left[1+\cos\left(b\nu\right)\right]/2$. Substituting Eq. (S20) into Eq. (S19) and noticing that

$$\mathrm{i}\left(\mathbb{U}\otimes\mathbb{U}^{*}\right)\otimes\mathbb{U}-H\mathbb{U}=\mathrm{i}\frac{\eta^{2}}{a^{2}}\mathbb{U}, \tag{S21}$$

we will have

$$\begin{aligned}\frac{\partial \eta}{\partial Z}Qe^{\mathrm{i}\phi}+\mathrm{i}\frac{\partial \phi}{\partial Z}\eta Qe^{\mathrm{i}\phi}&=\mathrm{i}\frac{\eta^{2}}{a^{2}}\eta Qe^{\mathrm{i}\phi}-\mathrm{i}\Delta\eta Qe^{\mathrm{i}\phi}-\eta Qe^{\mathrm{i}\phi}\\&+\left[\mathrm{i}C^{*}\eta^{2}e^{\mathrm{i}2\phi}Q\otimes Q+\mathrm{i}C^{2}\eta Qe^{-\mathrm{i}\phi}+\mathrm{i}2C\eta^{2}Q\otimes Q\right]+\mathrm{i}2\left|C\right|^{2}\eta Qe^{\mathrm{i}\phi}\end{aligned} \tag{S22}$$

The terms enclosed in square brackets represent parametric mixing between the soliton and the homogenous background. Under the small-perturbation assumption, one balance is achieved between SPM and gate spectral filtering, while the other is achieved between parametric gain and uniform loss. The frequency-dependence of parametric gain can then be neglected in approximate analysis. Thus, we assume $Q\otimes Q\approx kQ$ where the constant $k=0.687$ can be retrieved with numerical curve fitting. By separating the real and imagery terms of Eq. (S22), we obtain the following coupled equations

$$\frac{\partial \eta}{\partial Z}=-\eta+k\left|C\right|\eta^{2}\sin\left(\phi-\phi_{C}\right)+\left|C\right|^{2}\eta\sin\left[2\left(\phi-\phi_{C}\right)\right], \tag{S23a}$$

$$\frac{\partial \phi}{\partial Z}=-\Delta+\frac{\eta^{2}}{a^{2}}+3k\left|C\right|\eta\cos\left(\phi-\phi_{C}\right)+\left|C\right|^{2}\cos\left[2\left(\phi-\phi_{C}\right)\right]+2\left|C\right|^{2}, \tag{S23b}$$

where $\phi_C = \arg(C)$.

When the phase detuning $\Delta \gg 1$, the lower-branch homogenous solution $C \approx -\mathrm{i}S/\Delta$. Further neglecting the higher-order terms with $|C|^2$, we get

$$\frac{\partial \eta}{\partial Z} \approx -\eta + \frac{kS}{\Delta}\eta^2 \cos\phi, \tag{S24a}$$

$$\frac{\partial \phi}{\partial Z} \approx -\Delta + \frac{1}{a^2}\eta^2 - \frac{3kS}{\Delta}\eta \sin\phi. \tag{S24b}$$

A fix-point solution of Eq. (S24) reads

$$\eta \approx a\sqrt{\Delta}, \tag{S25a}$$

$$\phi \approx \arccos\left(\frac{\sqrt{\Delta}}{akS}\right). \tag{S25b}$$

A comparison between the numerical results and the approximate analytical results is shown in Fig. S3, showing relatively good agreement. The parameters are $\Delta = 30$ and $S^2 = 60$.

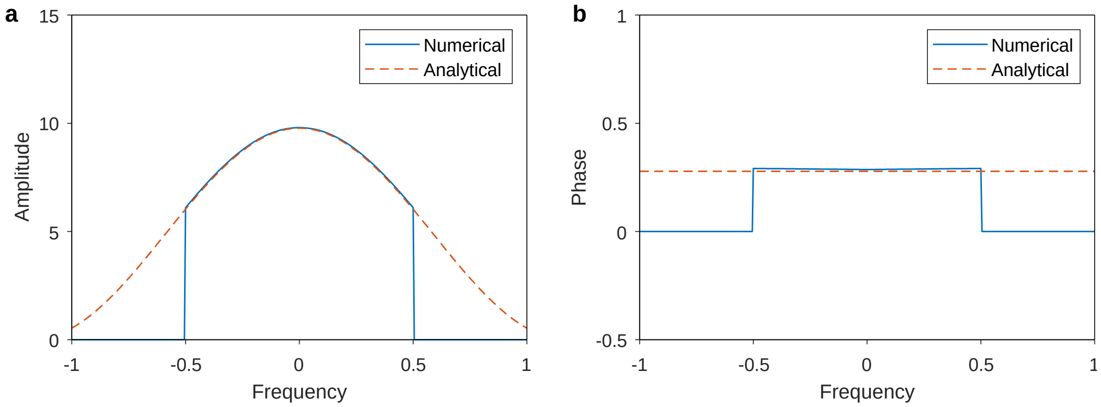

Fig. S3. Comparison between numerical and approximate analytical results for the Nyquist cavity soliton. a Spectral amplitude. **b** Spectral phase. The simulation parameters are $\Delta = 30$ and $S^2 = 60$.

From Eq. (S25a), we find that the soliton phase shifting rate (the imaginary eigenvalue $\xi = \eta^2/a^2$) is clamped by the pump-cavity phase detuning (Δ), i.e., $\xi = \Delta$. This conclusion is identical to that for the conventional cavity solitons[S4], and can be easily understood by noticing the fact that the soliton phase-synchronized with the

external pump will get the maximum gain when it is coherently combined with the pump field. It is noted that the analytical approximation by Eq. (S25) is more accurate with lower pump intensity under the premise of soliton sustainment. With the increase of pump power, the cavity soliton will show slightly increasing distortions in comparison to the eigenfunction, as is indicated by Fig. 4**e** of the main paper.

The soliton stability can be investigated according to Eq. (24). Figure S4 shows one simulated example in which the initial soliton parameters deviate from the stationary solution. With the increase of propagation distance, the soliton finally converges to the fixed point.

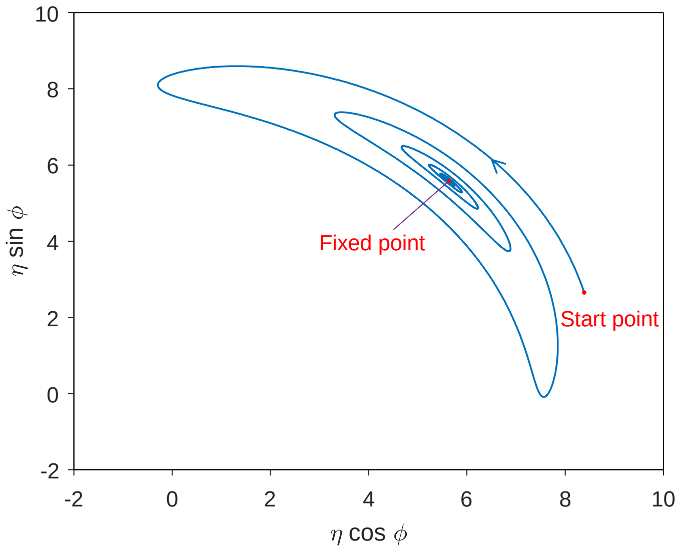

Fig. S4. Dynamic evolution of Nyquist cavity soliton. The simulation parameters are $\Delta = 30$ and $S^2 = 60$.

1.4 Nyquist soliton molecules

Depending the initial field (e.g., when the pulse duration is much wider than the transform-limited value), stable soliton molecules composed of multiple closely bound pulses can also be observed in simulations. These Nyquist soliton molecules are also related to the eigenfunctions of combined SPM and gate spectral filtering. Figure S5 shows the numerical results simulated based on Eqs. (S5)-(S7). The parameters are $\xi = \Delta = 50$ and $S^2 = 60$. Figure 3 of the main paper shows experimental evidences of Nyquist soliton molecules and their transitioning in a pulse pumped fiber ring cavity.

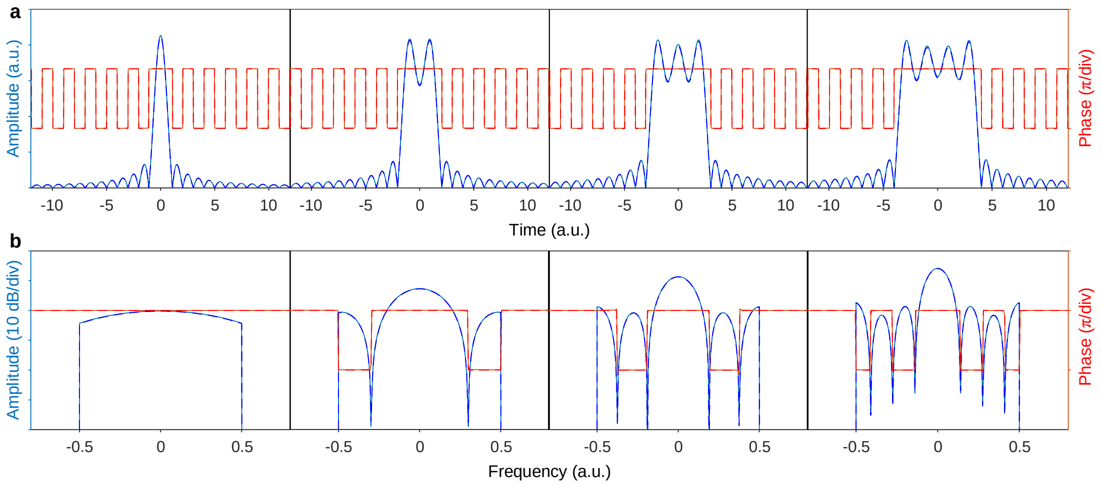

Fig. S5. Compact Nyquist soliton molecules. a Pulse shape. **b** Spectrum. From left to right, the molecules contain 1, 2, 3 and 4 solitons, respectively. Solid: cavity soliton; dash: Kerr-and-filter eigen function. The solid and dash lines are overlaid and visually indistinguishable. The simulation parameters are $\xi = \Delta = 50$ and $S^2 = 60$.

1.5 Pulse pumped equation for the fiber cavities

When the Kerr cavity is pumped by optical pulses, a new term should be introduced to account for the desynchronization between the cavity soliton and the external pump pulse. The mean-field equation reads

$$\frac{\partial A}{\partial z} = -(\alpha + \mathrm{i}\delta)A + \mathrm{i}\gamma |A|^2 A - \rho\left(\mathrm{i}\frac{\partial}{\partial t}\right)^n A - \mathrm{i}\frac{\beta_2}{2}\frac{\partial^2}{\partial t^2}A - \zeta\frac{\partial}{\partial t}A + \kappa A_{\mathrm{p}} \tag{S26}$$

where β_2 is the second-order dispersion; $\zeta = d/\left[v_{\mathrm{g}}(1+d)\right]$ is the desynchronization induced temporal drifting; and $d = (R_{\mathrm{p}} - R_{\mathrm{c}})/R_{\mathrm{c}}$ represents the relative difference between the pump pulse repetition rate R_{p} and the cavity free spectral range R_{c}; v_{g} is the soliton group velocity.

For the simulation results shown in Figs. 2, 3 and 5 of the main paper, a simplified model is employed in which each effect is considered separately by a lumped term in one roundtrip. The field at the end of the (m+1)-th round is related to that after the m-th round by

$$A_{m+1} = \sqrt{(1-\theta)(1-\alpha_L)}e^{-\mathrm{i}(\delta_L + \gamma P_m L)} F A_m + \sqrt{\theta} A_{\mathrm{p}} \tag{S27}$$

where A is the field amplitude normalized such that $P = |A|^2$ represents the field power; θ is the pump-cavity power coupling ratio; α_L is the uniform roundtrip power

loss; δ_L is the pump-cavity phase detuning; γ is the average Kerr coefficient; L is the roundtrip length; and A_{p} is the pump field. The operator F accounts for the effects that can be easily applied in the frequency domain, including the frequency-dependent filtering loss, the group velocity dispersion and the pump-cavity desynchronization; i.e.

$$\mathcal{F}\left\{FA_m\right\} = H_{\mathrm{T}}\mathbb{A}_m e^{\mathrm{i}d_2\omega^2/2} e^{-\mathrm{i}\omega\tau} \quad \text{(S28)}$$

where $\mathbb{A}_m = \mathcal{F}\left\{A_m\right\}$ is the field spectrum; H_{T} represents the transfer function of the spectral filter; d_2 is the roundtrip group delay dispersion; τ is the desynchronization induced time shift between the cavity soliton and the pump pulse; and ω is the angular frequency. Note that calculating Eq. (S27) is actually equivalent to integrating Eq. (S26) with the split-step Fourier method with a step size equal to one roundtrip. The parameters are related by

$$\theta = \kappa^2 L^2 \,, \; \alpha_L = 1 - e^{-2\alpha L} \,, \; \delta_L = \delta L \,, \; d_2 = \beta_2 L \,, \; \tau = \zeta L \,. \quad \text{(S29)}$$

2. Experiments

2.1 Fiber cavity stabilization

The detailed experimental setup is shown in Fig. S6. To maintain a stable pump-cavity frequency detuning for soliton generation, the laser frequency is first locked to the fiber cavity resonance by sending a probe light in the backward direction and using the Pound-Drever-Hall locking technique. An acousto-optic frequency shifter is then used to tune the frequency of the pump pulse in the forward direction. The polarizations of the probe and the pump are adjusted orthogonal to each other to minimize their mutual interference.

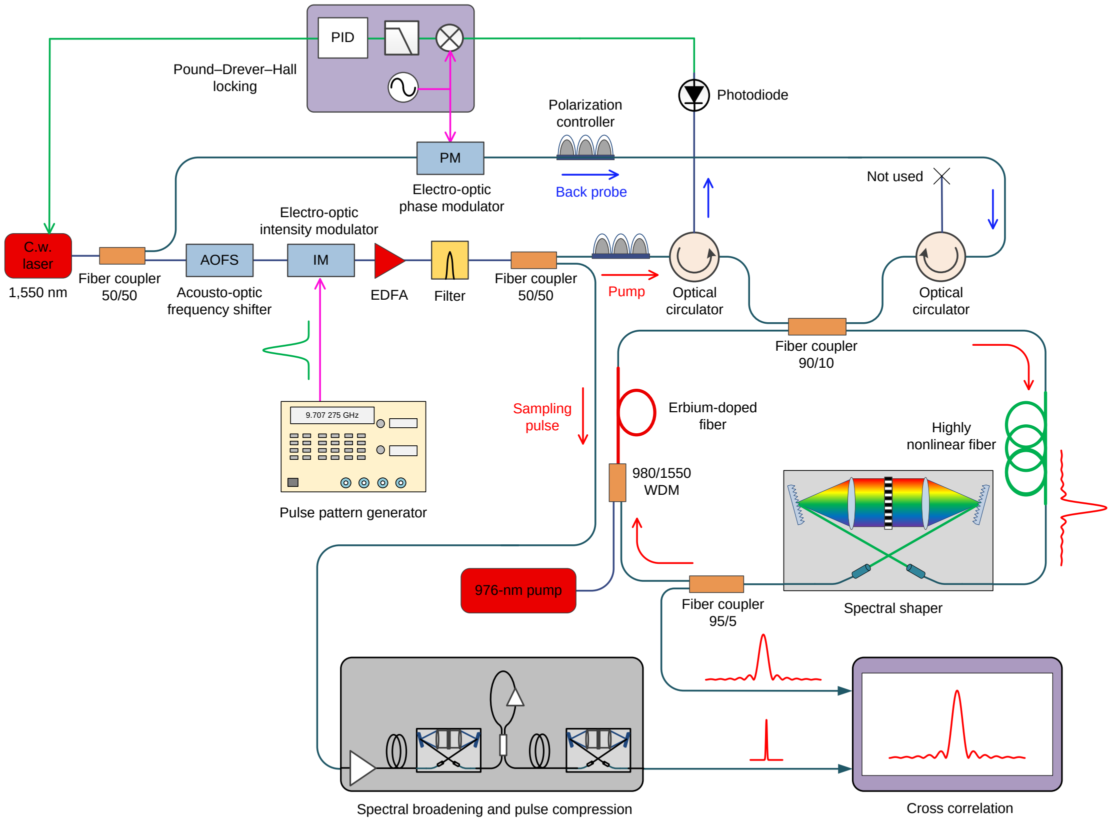

Fig. S6. Experimental setup for soliton generation and characterization. PID: proportional–integral–derivative controller; EDFA: erbium-doped fiber amplifier; WDM: wavelength-division-multiplexed coupler.

2.2 Intensity cross correlation for soliton characterization

The intensity cross correlation setup for measuring the soliton pulse shape is shown in Fig. S7. The synchronous sampling pulse is generated through a multiple-stage spectral broadening and pulse compression procedure. A portion of the Gaussian pump pulse is first amplified to a peak power of ~200 W, and sent through a 50-m highly nonlinear fiber (HNLF). The spectrum is broadened to ~2 nm and the pulse is compressed by a spectral shaper. A nonlinear amplifying loop mirror (NALM) composed of 2-m HNLF and 0.5-m Erbium-doped fiber (LIEKKI Er-110) is used to improve the pulse quality and further broaden the spectrum to ~6 nm. After passing through another 50-m HNLF, the spectral bandwidth exceeds 100 nm. A second spectral shaper is used to select the spectrum within 1527-1567 nm and shape the pulse to a Gaussian function with a full-width-at-half-maximum of 0.2 ps. The peak power of the output pulse is ~130 W. The pulse is much narrower than the solitons to be measured, making it possible to capture the fine temporal features with a high resolution.

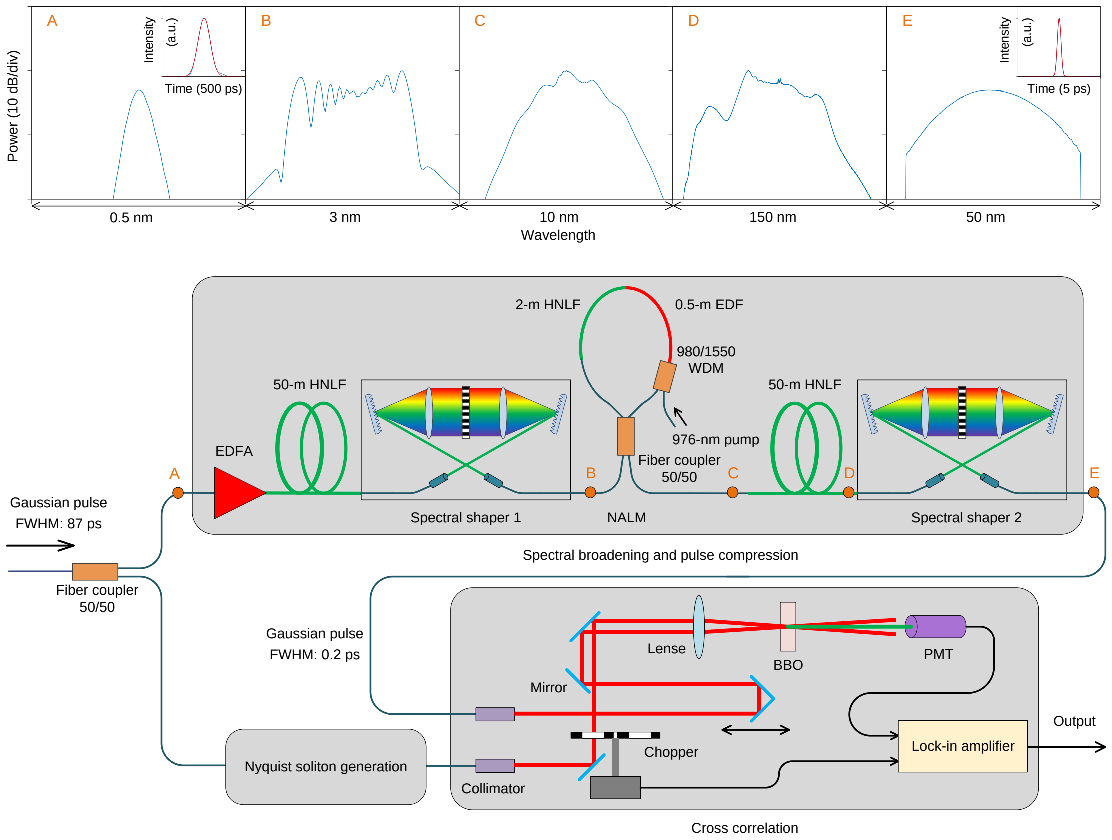

Fig. S7. Intensity cross correlator for soliton characterization. The insets on top show the spectra at different locations (A, B, C, D, E). The pulse waveform at point A detected by a high-speed photodiode and the autocorrelation of the compressed Gaussian pulse at point E are also shown (blue: measured; red: calculated). FWHM: full-width-at-half-maximum; EDFA: Erbium-doped fiber amplifier; HNLF: highly nonlinear fiber; EDF: Erbium-doped fiber; WDM: wavelength-division-multiplexed coupler; NALM: nonlinear amplifying loop mirror; BBO: beta barium borate crystal; PMT: photomultiplier tube.

2.3 Nyquist soliton transition

Figure S8 shows the full data of the soliton transition process in Fig. 3 of the main paper. With the increase of pump frequency detuning from s_{16} to s_1, the soliton molecule pulse width decreases from ~126 ps to ~4 ps and the spectral sidelobes disappear pair by pair. A single Nyquist soliton with a smooth spectrum is finally formed.

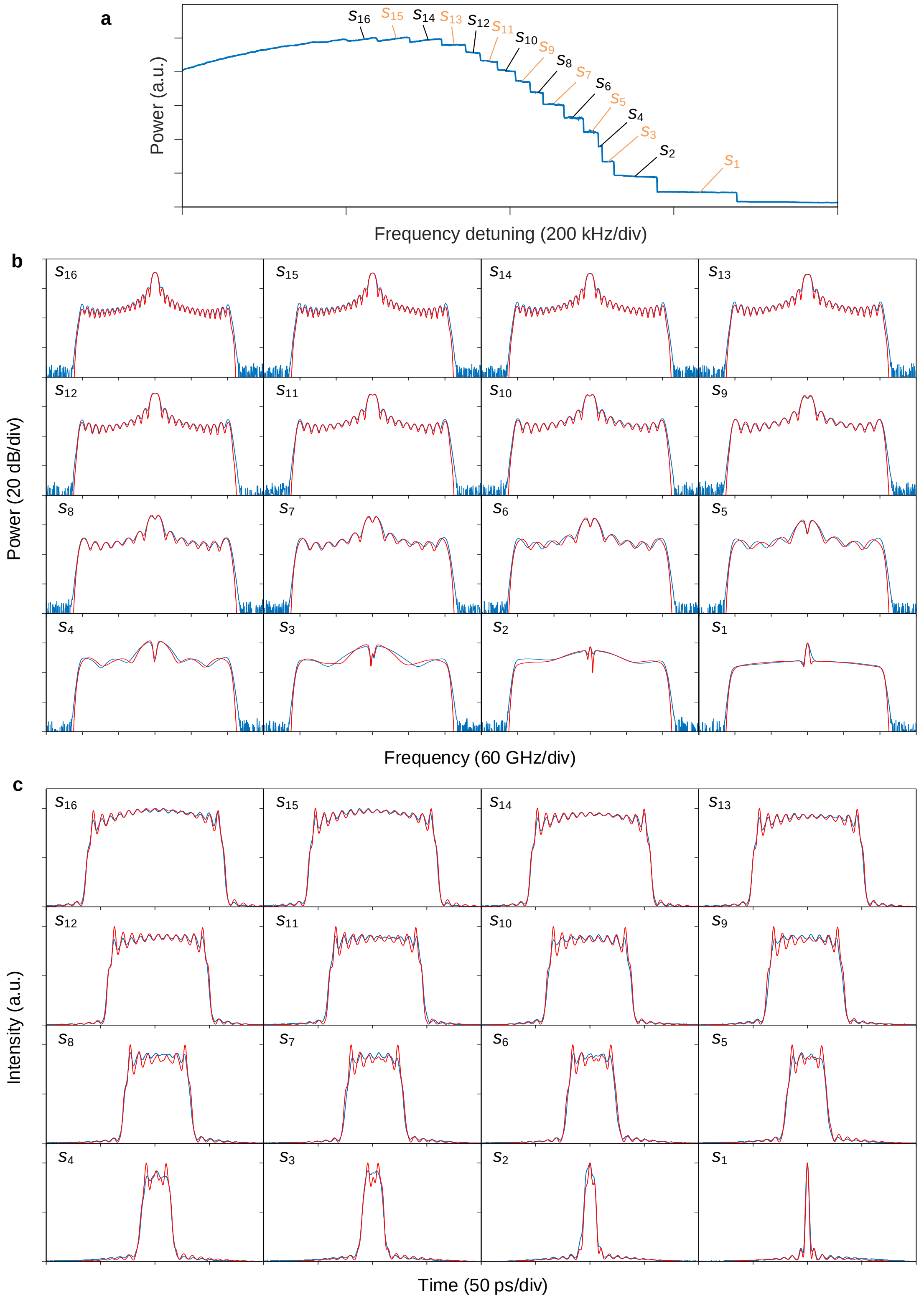

Fig. S8. Nyquist soliton transition under pulsed pumping. a Intracavity power versus pump frequency detuning. **b,c** Spectrum and pulse shape at the different steps marked in **a** (s_{16} to s_1). Blue: measured; red: simulated.

2.4 Effect of pump-cavity desynchronization

It is found that the soliton transition process is affected by the desynchronization between the pump pulse and the cavity. Figure S9**a** shows multiple intracavity power traces measured for slightly different pump pulse repetition rates. The simulation results are shown in Fig. S9**b**. The measured filter transfer function shown in Fig. 2**c** of the main paper is employed in the simulation. The desynchronization parameter (i.e., relative difference between the pump pulse repetition rate and the cavity FSR) varies between $\pm 3\times10^{-4}$. The widest single-soliton step is achieved when the desynchronization is slightly negative (of order of 10^{-5}). When the desynchronization magnitude gets larger, the single-soliton region becomes narrower and may even disappear. For large desynchronization, the intracavity field drops to the lower-branch homogenous state before reaching the single-soliton state. Simulations show that the soliton transition behavior may also be affected by the pump pulse shape as well as the asymmetry of the filter function.

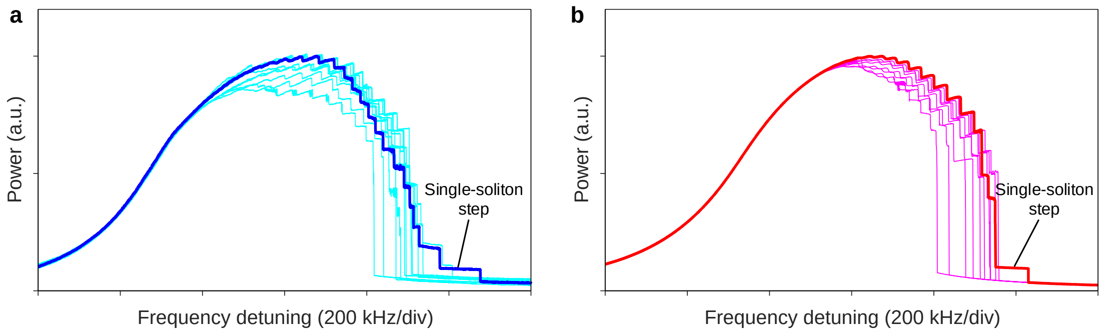

Fig. S9. Multiple intracavity power traces with slightly different pump repetition rates. a Measured. **b** Simulated. One trace showing single soliton generation is highlighted.

2.5 Soliton evolution with pump power

Figure S10**a** shows the overlaid soliton pulses corresponding the states of II, IV and V indicated in Fig. 4**a** of the main paper. The detuning is fixed at 20 and the pump power is 40 (II), 250 (IV) and 540 (V), respectively. It can be observed that, with the increase of pump power, the main pulse width keeps nearly unchanged while the oscillating tails get more prominent. The rise of the oscillating tails is responsible for the change of the spectrum shown in Fig. 4**e**. As explained in the main paper, the high

pump power breaks the (approximately) independent balance between parametric gain and uniform loss. The soliton pulse shape thus changes adaptively to achieve a new composite balance as illustrated in Fig. S10**b**.

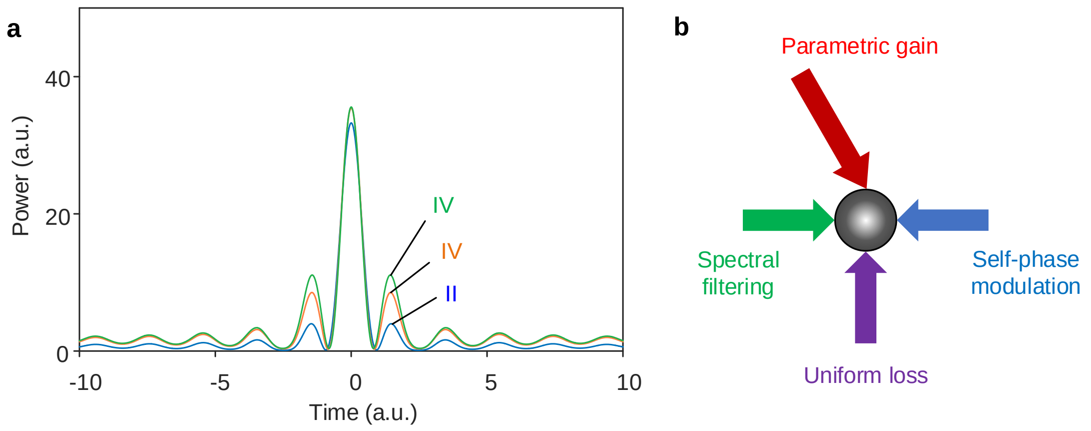

Fig. S10. Solitons under different pump power. a Overlaid pulse waveforms. **b** Composite balance achieved when the pump power is much higher than the minimum value required for soliton sustainment.

2.6 Spectral evolution in one roundtrip

Figure S11 shows the soliton spectra measured before and after the spectral shaper. The uniform insertion loss of the spectral shaper has been subtracted. No significant change can be observed. The flat pedestal in the spectrum afore spectral shaper, which is more than 20 dB lower than the soliton spectral intensity, is mainly attributed to the amplifier spontaneous emission noise.

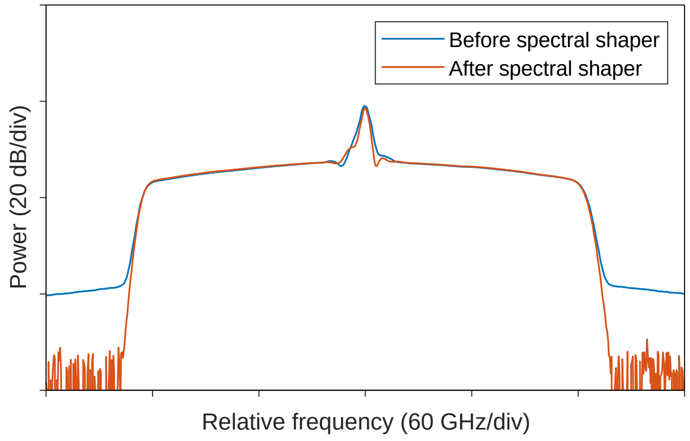

Fig. S11. Soliton spectra before and after the spectral shaper.

2.7 Numerical simulation of dispersion-less soliton microcomb

In this section, we show numerical simulation results of filter-driven dispersion-less solitons generated within on-chip integrated microresonators. Figure S12**a** shows two conceptual designs including ring and Fabry–Pérot structures. For the ring cavity, spectral control is integrated through a periodic Bragg boundary that is implemented on the inner side of the ring waveguide. Therefore, the frequency components falling within the stopband can be confined in the cavity while the out-of-band frequencies experience high loss and will leak out. For the Fabry–Pérot cavity, the same goal can be accomplished by tailoring the two Bragg reflectors. Moreover, the photonic crystal structure provides a new degree of freedom for dispersion engineering to achieve negligible dispersion[S5].

The simulation is performed based on the following equation

$$\begin{aligned}\frac{\partial A}{\partial z} = -(\alpha + \mathrm{i}\delta)A + \mathrm{i}\gamma\left(|A|^2 - f_{\mathrm{R}}\tau_{\mathrm{R}}\frac{\partial |A|^2}{\partial t}\right)A - \rho\left(\mathrm{i}\frac{\partial}{\partial t}\right)^n A \\ -\mathrm{i}\left(\frac{\beta_2}{2}\frac{\partial^2}{\partial t^2} + \mathrm{i}\frac{\beta_3}{6}\frac{\partial^3}{\partial t^3}\right)A - \zeta\frac{\partial}{\partial t} + \kappa A_{\mathrm{p}}\end{aligned} \tag{S30}$$

To obtain results more compatible with experiments, the third-order dispersion and Raman effect are also included in the model. The typical parameters of silicon nitride microresonators are employed and summarized in Table 1.

The comb line spacing (R_{c}) is 50 GHz corresponding to a cavity roundtrip length (L) of 3 mm. The pump field is generated by modulating the phase and amplitude of a continuous-wave laser, given by

$$A_{\mathrm{p}} = \sqrt{P_{\mathrm{in}}}\cos\left[\frac{m_i}{2}\cos\left(2\pi R_{\mathrm{p}}t\right) - \frac{\pi}{4}\right]\exp^{\mathrm{i}m_{\mathrm{p}}\cos\left(2\pi R_{\mathrm{p}}t\right)} \tag{S31}$$

where $P_{\mathrm{in}} = 0.2$ W is the pump power; $m_{\mathrm{i}} = \pi/2$ is the intensity modulation index; $m_{\mathrm{p}} = \pi$ is the phase modulation index; R_{p} is the modulation frequency related to the desynchronization parameter ζ by $\left(1/R_{\mathrm{c}} - 1/R_{\mathrm{p}}\right)/L$. Here, pump phase modulation is employed to trap the soliton around the maxima of the pump intensity[S7].

Figure S12**b** shows the single soliton generated when the pump phase detuning is gradually increased from $-1\ \mathrm{m}^{-1}$ to $13.3\ \mathrm{m}^{-1}$ (i.e., scanning the pump laser frequency

from the blue-detuned side to the red-detuned side as in usual experiments[S8]). The full-width-at-half-maximum is 74 fs. The comb spectrum is shown in Fig. S12**c**. There are ~250 lines spanning over 100 nm, within a 5-dB intensity range excluding the pump. The tilt of spectral envelop towards longer wavelength is attributed to Raman induced self-frequency shift[S9].

Table 1. Simulation parameters for soliton microcomb generation

Symbol	Definition	Value
α	Uniform loss[a]	$2.44\times10^{-3}\ \mathrm{m}^{-1}$
δ	Phase detuning	$13.3\ \mathrm{m}^{-1}$
γ	Kerr nonlinearity	$1\ \mathrm{W}^{-1}\mathrm{m}^{-1}$
f_R	Raman fraction	0.2
τ_R	Raman shock time	20 fs
n	Filter order	10
ρ	Frequency-dependent loss[b]	$2.60\times10^{-138}\ \mathrm{m}^{-1}\mathrm{Hz}^{-10}$
β_2	Second-order dispersion[c]	$1.70\times10^{-27}\ \mathrm{s}^2\mathrm{m}^{-1}$
β_3	Third-order dispersion	$-3.30\times10^{-42}\ \mathrm{s}^3\mathrm{m}^{-1}$
ζ	Desynchronization	$1.20\times10^{-16}\ \mathrm{s\ m}^{-1}$
κ	Pump coupling coefficient	$16.5\ \mathrm{m}^{-1}$

a. The corresponding loaded quality factor is 5×10^6.

b. $\rho=\alpha(\pi B)^{-n}$ where $B=10$ THz is filter bandwidth.

c. The dispersion coefficients are calculated around 1550 nm with the *COMSOL* software. The silicon nitride waveguide cross-section is $2\ \mu\mathrm{m}\times0.75\ \mu\mathrm{m}$. The refractive index profiles of silicon nitride and cladding silicon oxide are the same as in ref. S6. The resulting second-order dispersion is weakly normal.

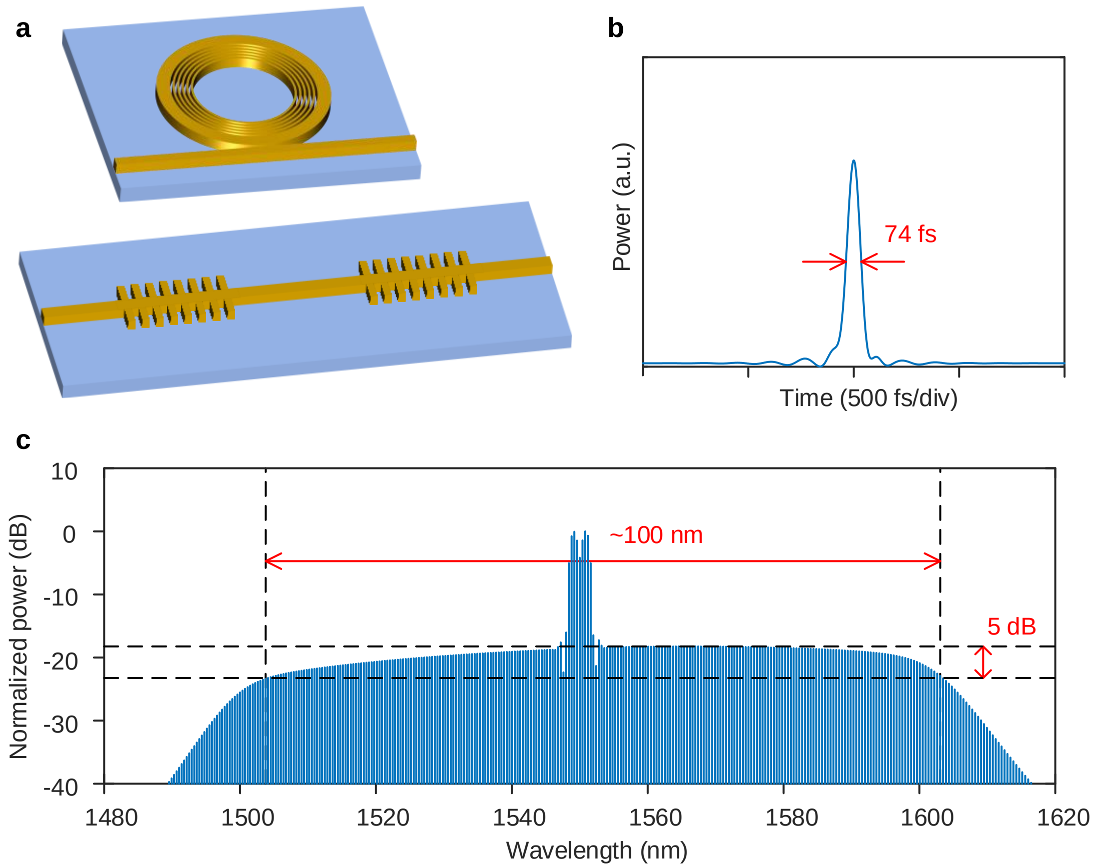

Fig. S12. Dispersion-less soliton microcomb generation with integrated microresonator. a Two conceptual designs of spectrally confined microresonator. Upper: ring; lower: Fabry–Pérot. **b** Femtosecond soliton pulse. **c** Comb spectrum.

References for Supplementary Materials

S1. Huang, S. W., Zhou, H., Yang, J., McMillan, J. F., Matsko, A., Yu, M., Kwong, D.-L., Maleki, L. & Wong, C. W. Mode-locked ultrashort pulse generation from on-chip normal dispersion microresonators. *Phys. Rev. Lett.* **114**, 053901 (2015).

S2. Spiess, C., Yang, Q., Dong, X., Bucklew, V. G. & Renninger, W. H. Chirped dissipative solitons in driven optical resonators. *Optica* **8**, 861–869 (2021).

S3. Perego, A. M., Mussot, A. & Conforti, M. Theory of filter-induced modulation instability in driven passive optical resonators. *Phys. Rev. A* **103,** 013522 (2021).

S4. Wabnitz, S. Suppression of interactions in a phase-locked soliton optical memory. *Opt. Lett.* **18,** 601–603 (1993).

S5. Yu S., Jung, H., Briles, T. C., Srinivasan, K. & Papp, S. B. Photonic-crystal-reflector nanoresonators for Kerr-frequency combs. *ACS Photon.* **6,** 2083–2089

(2019).

S6. Xue, X., *et al.* Thermal tuning of Kerr frequency combs in silicon nitride microring resonators. *Opt. Express* **24,** 687–698 (2016).

S7. Jang, J. K., Erkintalo, M., Coen, S & Murdoch, S. G. Temporal tweezing of light through the trapping and manipulation of temporal cavity solitons. *Nature Commun.* **6,** 7370 (2015).

S8. Herr, T., Brasch, V., Jost, J. D., Wang, C. Y., Kondratiev, N. M., Gorodetsky, M. L. & Kippenberg, T. J. Temporal solitons in optical microresonators. *Nature Photon.* **8,** 145–152 (2014).

S9. Karpov, M., *et al.* Raman self-frequency shift of dissipative Kerr solitons in an optical microresonator. *Phys. Rev. Lett.* **116,** 103902 (2016).
